# A Role for Mast Cell-Mediated Antibodies in the Formation of Cholesteatoma and Cholesteatoma-Induced Bone Erosion

**DOI:** 10.3390/diagnostics13030455

**Published:** 2023-01-26

**Authors:** Çiğdem Özdemir, Selçuk Kuzu, Yiğit Şenol, Tuba Yiğit, Erol Güldün, Abdulkadir Bucak, Şahin Ulu, Çiğdem Tokyol

**Affiliations:** 1 Department of Pathology, Faculty of Medicine, Afyonkarahisar Health Sciences University, Afyonkarahisar 03030, Turkey; 2 Department of Otorhinolaryngology, Faculty of Medicine, Afyonkarahisar Health Sciences University, Afyonkarahisar 03030, Turkey; 3 Department of Public Health, Faculty of Medicine, Afyonkarahisar Health Sciences University, Afyonkarahisar 03030, Turkey

**Keywords:** cholesteatoma, CD117, CD34, Ki67, chymase, tryptase, bone erosion

## Abstract

The study aimed to evaluate the effects and relationships between mast cells in the matrix, mast cell enzymes tryptase and chymase, epithelial proliferation, microvascular density, and bone destruction in cholesteatoma. Thirty-five biopsies diagnosed with cholesteatoma and seven healthy skin tissues taken from the retro-auricular region for control were evaluated. Immunohistochemical studies were performed with CD117, CD34, Ki-67, chymase, and tryptase antibodies, in a single session for all cases and the control group. The relationship between erosion size and antibody load was determined. The mean cholesteatoma epithelium Ki-67 was higher than the control group (*p* < 0.001). CD117-positive mast cells, chymase-positive mast cells, tryptase-positive mast cells, and microvessel density were significantly higher in the cholesteatoma matrix compared to the control group (*p* < 0.002, *p* < 0.001, *p* < 0.005). In the group with bone erosion scores of two and above, immunohistochemical markers tended to be higher. A positive correlation was found between CD117 and chymase, tryptase, and microvessel density; between tryptase, chymase, and microvessel density; and between chymase and microvessel density. CD117-positive mast cells and chymase-positive mast cells stimulate angiogenesis, increase the epithelium’s proliferative capacity in the cholesteatoma matrix, and form cholesteatoma. The increased proliferation of cholesteatoma epithelium and increased vascular density in the matrix exacerbate bone erosion.

## 1. Introduction

Cholesteatoma is the accumulation of keratinised squamous epithelium in the middle ear. It is important to note that cholesteatoma, despite its benign nature, can cause several complications inside the skull, including hearing loss, facial nerve paralysis, and various complications associated with blood vessels [1]. Even though its incidence is not known with certainty, it occurs in approximately 6 out of every 100,000 individuals. Three types have been defined: congenital, acquired, and unclassifiable [2]. Histomorphology consists of three layers: a cystic part, a matrix, and a perimatrix. A cystic part consists of dead keratinocytes. The matrix layer consists of the stratified keratinised epithelium; this epithelium has a high proliferation capacity. The stroma is referred to as the perimatrix layer; this stroma consists of connective tissue, vessels, polymorphous leukocytes, macrophages, and mast cells [3,4]. The etiopathogenesis of cholesteatoma is still controversial, and its development’s cellular and molecular mechanisms are still unclear [5]. However, it has been demonstrated that the matrix and perimatrix are involved in developing destructive cholesteatoma. The increased proliferation of keratinised epithelium facilitates bone erosion by promoting the development and progression of cholesteatoma [6,7].

Ki-67 is a nuclear-expressed marker, indicating the proliferative capacity of cancer cells, and is used as a prognostic indicator in most cancers [8,9]. The Ki-67 index in the cholesteatoma matrix is higher than in normal skin tissue [10].

Angiogenesis has been identified in many physiological and pathological conditions and plays an important role. In addition, angiogenesis is becoming a target in cancer treatment, and antiangiogenic drugs are currently being developed [11]. A common misconception is that angiogenesis is controlled solely by endothelial cells, whereas stroma, stromal fibroblast, and inflammatory cells play significant roles as well. The stromal fibroblasts and inflammatory cells in the stroma regulate angiogenesis by secreting cytokines and proangiogenic factors, including Bv8/PROK2, which belongs to the PDGF family, FGF family, VEGF family, and angiopoietin family [10,12,13,14]. Cholesteatoma stroma is highly vascularised [11,15]. The most common method for the semi-quantitative assessment of angiogenesis in both benign and malignant conditions is the measurement of microvessel density (MVD) using endothelial markers. It is even considered one of the most useful prognostic markers. To evaluate and measure MVD, the immunohistochemical method and endothelial markers such as CD31, CD34, and CD105 are used most frequently [16,17]. There are a limited number of studies examining MVD in cholesteatoma [18]. Previous studies have reported detecting inflammatory cells, leucocytes, monocytes, mast cells, and macrophages in cholesteatoma [15,19]. Mast cells have been studied recently as central cells involved in tissue remodelling and development and induction of an immune response [20]; we also know that immunotherapy is an essential target in treating solid organ tumours.

Initially derived from bone marrow progenitor cells, mast cells mature in response to the microenvironment within tissues [21]. Mast cells contain potent biologically active molecules in their granules. These molecules are released when mast cells are stimulated. Thanks to these molecules, mast cells affect the functions of fibroblasts, endothelial cells, and smooth muscle cells. Mast cells can transfer the contents of their granules directly to endothelial cells [22]. In addition, mast cells synthesise and secrete angiogenic cytokines. Cytokines such as endothelial growth factor (VEGF), fibroblast growth factor-2 (FGF-2), tryptase and chymase, transforming growth factor beta (TGF-1), and nerve growth factor (NGF) are involved [23]. In particular, the mast cell enzyme tryptase is critical in regulating angiogenesis and the invasion of tumours [20]. Similarly, chymase is a mast cell-released protease enzyme involved in wound healing, which has been associated with tumour angiogenesis [24]. A member of the tyrosine kinase receptor family, c-Kit or cluster of differentiation 117 (CD117) plays a key role in mast cell differentiation and function [25]. The c-Kit or cluster of differentiation plays a role in the process of angiogenesis in the subepithelial connective tissue of cholesteatoma [15]. It has been shown in previous studies that the cholesteatoma matrix contains an increased number of CD117-positive mast cells [26]. However, the function of mast cells within the matrix has yet to be fully understood [4].

This study examined the effects and relationships between mast cells in the matrix, mast cell enzymes tryptase and chymase, epithelial proliferation, microvascular density, and bone destruction in cholesteatoma, the pathogenesis of which is still controversial but has an aggressive course, like solid organ cancers.

## 2. Materials and Methods

This study included 35 biopsies diagnosed with cholesteatoma in Afyonkarahisar Health sciences University Faculty of Medicine, Department of Medical pathology, between 1 January 2014 and 1 January 2017. Seven normal skin tissues obtained from the retro-auricular region were taken as the control group. The age, gender, and clinical data of the patients were ascertained from the hospital information system. Haematoxylin-eosin (HE) stained preparations made from the tissues by embedding them in paraffin after 10% formaldehyde fixation were examined. Immunohistochemical studies were performed with CD117, CD34, Ki-67, chymase, and tryptase antibodies, in a single session for all cases and the control group. We used such a method to ensure standardisation during immunohistochemical staining. For each antibody in the study, four micron-thick sections from the tissues in the paraffin blocks were included in the research, and we took the control group on poly-L-lysine-coated slides. In the IHC studies, the antigen retrieval technique was used, and the avidin–biotin–peroxidase complex method was applied. The antibodies were examined in a Leica band max automatic immunohistochemical device. The Bond Polymer Refine Detection kit (DS9800, Leica Biosystems, Buffalo Grove, IL) was used for each antibody. The necessary staining procedure on the data sheet was applied, and appropriate positive and negative controls were used for each antibody. The characteristics of the primary anticipation used in the immunohistochemical study are listed in Table 1. The prepared samples were examined under an Olympus BX51 model microscope after the coverslip was covered with the ultra-mount.

### 2.1. Immunohistochemical Assessment

To determine the amount of microvessel density, CD117-positive mast cells, tryptase-positive mast cells, and chymase-positive mast cells in cholesteatoma and control skin tissue, we first determined the densest area in three areas with low power magnification (×100 and ×200) in each biopsy. Then 4–5 fields at a magnification of ×400 under an ocular grid (0.075 mm^2^) were counted. Then, the average value in each mm^2^ area was determined with the following formula (Number of cells or microvessel per mm^2^ = Number of cells or microvessel × magnification of 400/Area of Ocular Grid (0.075 mm^2^). Formula validated in Sharma and colleagues‘ study [27].

Any endothelial lined-up vessel lumen or endothelial cell cluster appearing brown and separate from an adjacent cluster was a single countable microvessel.

For Ki-67 at ×400 magnification, each biopsy’s cholesteatoma epithelium and the control skin tissue epithelium were the most intense. We calculated the number of positive cells among these cells by counting 200 cells [28].

### 2.2. The Assessment of Temporal Bone and Ossicular Erosion

The assessment of temporal bone and ossicular erosion was based on the operative notes and the preoperative CT scans. For the quantification of ossicular erosion, the operated ears were classified according to a modification of the STAMCO classification [22]: 0 (no ossicles eroded), 1 (one ossicle eroded), 2 (two ossicles eroded) or 3 (three ossicles eroded).

### 2.3. Statistical Assessment

Obtained data were evaluated with descriptive statistics (arithmetic mean, standard deviation, percentage distributions, and minimum and maximum values), and they were assessed with the Shapiro–Wilk test if continuous data did not fit normal distribution (*p* < 0.05). The Mann—Whitney U test was used to compare the paired groups. The Spearman Rank Differences Correlation Coefficient test was used to evaluate the correlation of two measurement data. Statistical significance level *p* was accepted as <0.05. Bonferroni-type adjustment was applied to reduce the chance of making a Type I error when multiple measures were tested. Adjusted *p* level (*p*_adjusted_) was calculated with formula “*p*/the number of comparisons”.

## 3. Results

The study included 35 patients, 25 (71.4%) of whom were males and 10 (28.6%) of whom were females. The mean age of the patients was 42.66 ± 16.61 (range 20–77).

**Figure 1:**
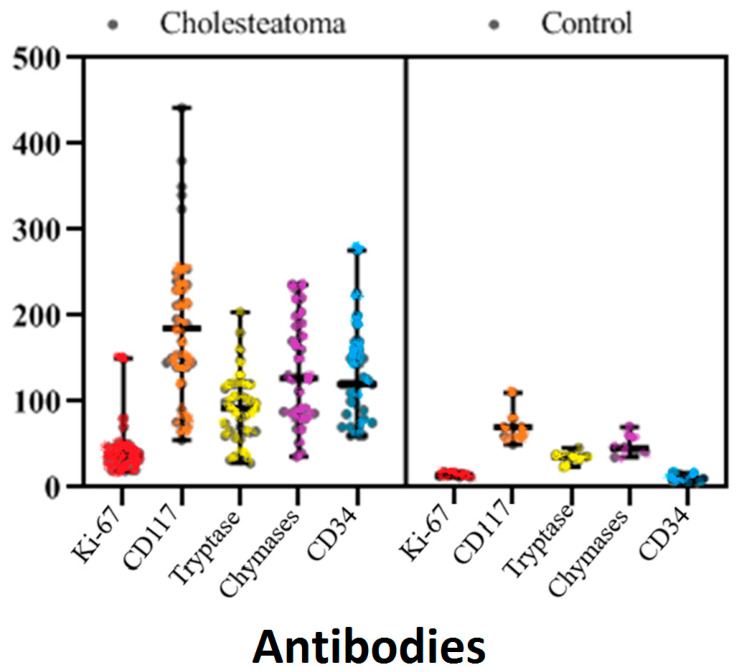
Kİ-67, CD117, tryptase, chymase, and CD34 distribution graph of the patient and control groups.

In comparing the immunohistochemical evaluations between the cholesteatoma and control groups: the mean Ki-67 detected in the epithelium in the control group was 14.43 ± 2.37 and the mean in the perimatrix in the cholesteatoma group was 41.23 ± 23.19. The Ki-67 proliferative index was significantly higher in the cholesteatoma epithelium compared to the epithelium of the control group (*p* < 0.001) (Table 1, Figure 1).

A comparison of CD117-positive mast cell count: the mean CD117 value was 71.43 ± 19.52/mm^2^ in the subepithelial area (dermis) in the control group, and the mean CD117 value was 194.80 ± 92.54/mm^2^ in the cholesteatoma matrix. The difference between the two groups regarding the mean of CD117-positive mast cells was statistically significant (*p* < 0.002).

Based on comparisons between chymase-positive mast cells and tryptase-positive mast cells in the subepithelial area and cholesteatoma matrix of the control group, our findings are as follows:

The mean of chymase-positive mast cells was 48.57 ± 12.25/mm^2^ in the control group and 133.14 ± 59.62/mm^2^ in the cholesteatoma matrix; the difference was statistically significant (*p* < 0.001) (Table 2, Figure 1).

The mean of tryptase-positive mast cells was 35.57 ± 6.68/mm^2^ in the control group and 91.89 ± 41.68/mm^2^ in the cholesteatoma matrix; the difference was statistically significant (*p* < 0.003).

Chymase-positive and tryptase-positive mast cells are more prevalent in the cholesteatoma matrix than in the control group. The mean number of chymase-positive mast cells was also dominant.

**Figure 2:**
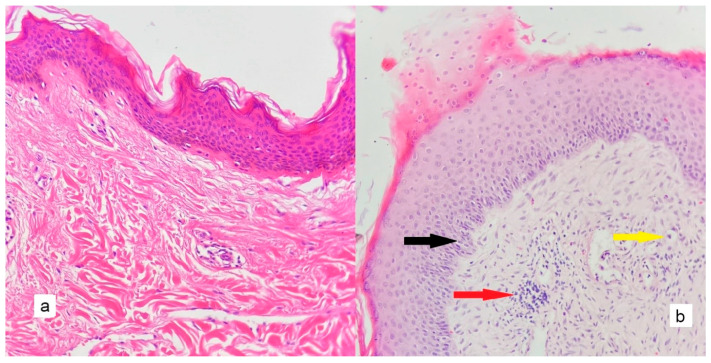
Compared to control tissue and cholesteatoma, there is an increased order in the cholesteatoma epithelium (black arrow), increased inflammatory cells in the stroma (red arrow), increased vascularity (yellow arrow), and a significant increase in connective tissue ((**a**): control tissue, (**b**): cholesteatoma tissue) (HEX200).

**Figure 3:**
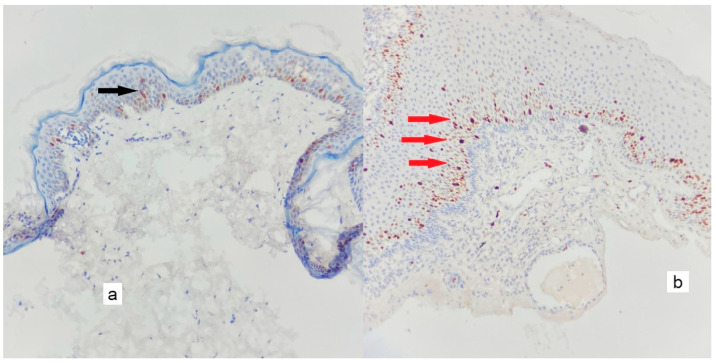
There are rare positive nuclear reactions observed only in the epithelium of the control tissue only at the basal layer when Ki-67 (black arrow) is immunohistochemically stained. In contrast, the cholesteatoma epithelium exhibits an intense nuclear reaction both at the basal layer and at the epithelium’s higher levels (red arrows) ((**a**): control tissues, (**b**): cholesteatoma tissues) (×200).

**Figure 4:**
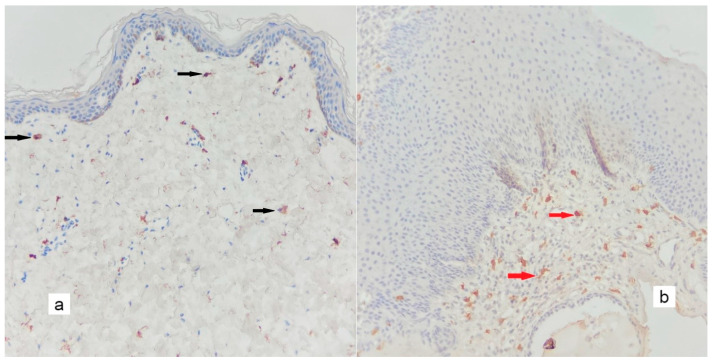
In CD117 immunohistochemical staining, a minimal number of positive mast cells are found in the stroma in the control tissue ((**a**), black arrows), whereas, in the cholesteatoma matrix ((**b**), red arrows), there is an increase in easy-to-identify positive mast cells (×200).

**Figure 5:**
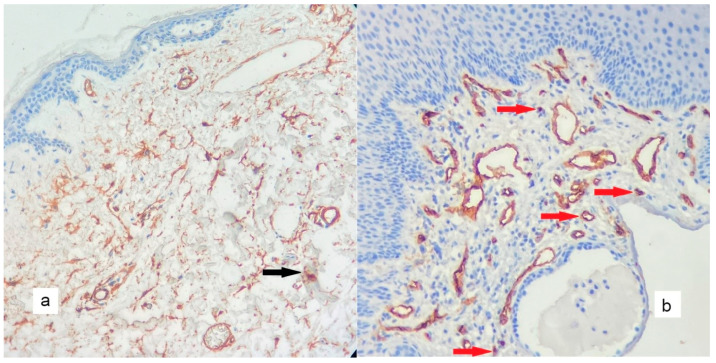
An increase in the number of small-diameter microvessels in the matrix of the cholesteatoma ((**b**), red arrows), when compared to the control tissue ((**a**), black arrow) (×200).

**Figure 6:**
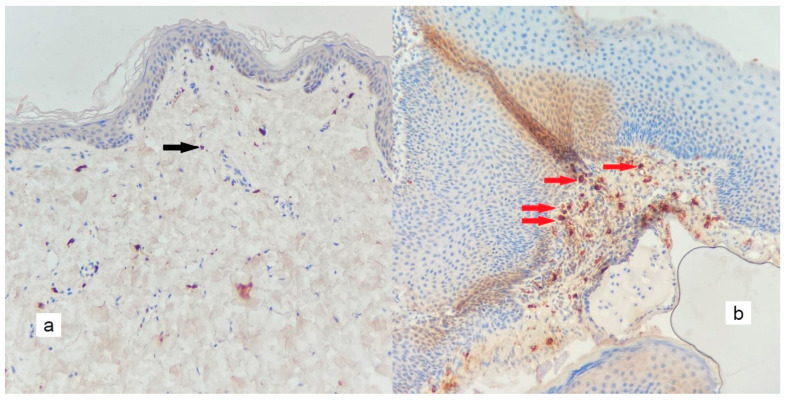
In chymase immunohistochemical staining, chymase-positive mast cells are sparsely positive in control tissue ((**a**), black arrow) and tend to aggregate in the cholesteatoma matrix ((**b**), red arrows) (×200).

**Figure 7:**
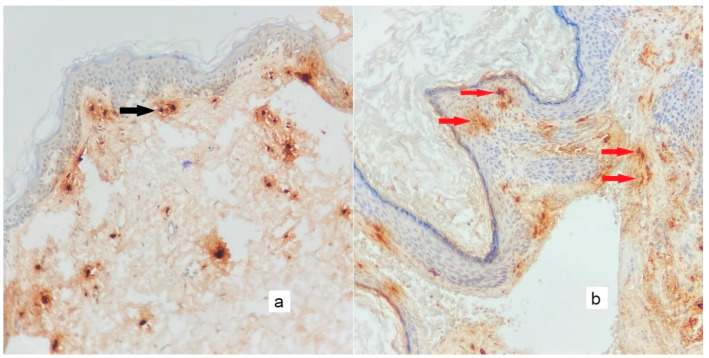
As seen in immunohistochemical staining with tryptase, mast cells are less prominent in the control tissue ((**a**), black arrow) and more prominent in the cholesteatoma matrix ((**b**), red arrows) (×200).

The mean MVD was significantly higher in cholesteatoma than in the control group (*p* < 0.005); the mean of the control group was 10.71 ± 4.15/mm^2^, while the mean of the cholesteatoma group was 123.26 ± 50.32/mm^2^. Figure 2, Figure 3, Figure 4, Figure 5, Figure 6 and Figure 7 compare the haematoxylin-eosin (HE) findings and immunohistochemical findings in control and cholesteatoma tissues.

For the quantification of ossicular erosion, the operated ears were classified according to a modification of the STAMCO classification; the distribution of our 35 cases was as follows:

The number of cases with a score of 0 (no bone erosion) was four (11.42%), the number of cases with a score of 1 (erosion in 1 bone) was five (14.28%), and the number of cases with a score of 2 (erosion in 2 bones) was twenty-six (74.28%). There was no case with a score of 3 (erosion in 3 bones). To perform group analysis, the 0 and 1 groups were combined into one "mild ossicular erosion" group, and the 2 and 3 groups were combined into one "advanced ossicular erosion” group. Accordingly, there was no statistically significant difference between the two groups in the Ki-67 index regarding the cholesteatoma epithelium, CD117-positive mast cells in the cholesteatoma matrix, tryptase-positive mast cells, chymase-positive mast cells, and microvessel density. Nevertheless, the mean of all studied immunohistochemical markers was higher in the group with a bone erosion score of 2 or above than in the other group. With increasing bone erosion scores, the means increased. (Table 3).

Assuming the correlation of immunohistochemical markers with each other: A positive correlation was found between CD117 and chymase (r = 0.993, *p* < 0.001), tryptase (r = 0.871, *p* < 0.001), and microvessel density (r = 0.775, *p* < 0.001). A positive correlation was found between tryptase and chymase (r = 0.837, *p* < 0.001) and microvessel density (r = 0.681, *p* < 0.001). A positive correlation was observed between chymase and microvessel density (r = 0.838, *p* < 0.001) (Table 4).

## 4. Discussion

Cruvelhhier first described cholesteatoma in 1829 as a pearl tumour based on its macroscopic appearance. First described in the 1800s, the pathogenesis of the destructive behaviour of this benign lesion remains unclear. In its modern definition, cholesteatoma is a cluster of keratins developed from keratinised squamous cell epithelium within the air cells of the temporal bone. Despite its beginning histological nature, cholesteatoma is hazardous because of intense inflammation and bone erosion in the surrounding bone tissue [15].

According to the literature, which is compatible with our study, most adult cholesteatoma cases appear in males in their 40s [29].

Cholesteatoma epithelium proliferative capacity, keratinocyte features, and intercellular interactions may contribute to the pathogenesis. A significant factor contributing to the destructive capacity of cholesteatoma is the proliferation capacity of the stratified squamous epithelium that forms the perimatrix [6]. Studies report Ki-67 (an essential marker for detecting proliferation in neoplastic or nonneoplastic epithelium) overexpression [7,26]. Similarly, in our study, the cholesteatoma epithelium’s proliferative capacity was considerably higher than that of normal skin.

The cholesteatoma matrix is similar to granulation tissue both structurally and in terms of cell diversity: the matrix consists of vessels, fibroblasts, and a wide variety of inflammatory cells [19]. The mast cells are among these: mast cells produce fibroblast growth factor, keratinocyte growth factor, and angiogenic and matrix remodelling factors [30]. In their investigation on acquired cholesteatoma, Hamed et al. found that basic fibroblast growth factors positively stained basal and parabasal keratinocytes. In addition, specific staining was observed in the basal columnar middle-ear epithelium and mast cell membrane [4]. Very few studies show the presence of mast cells in the cholesteatoma matrix. While mast cells have been demonstrated in previous studies to be present in cholesteatomas, the functions of these cells in developing cholesteatomas are unclear [31]. Our study, too, revealed a high number of mast cells in the cholesteatoma matrix compared to the control tissue.

Tryptase and chymase have been investigated and were found to be responsible for tumour angiogenesis [24]. When tryptase is secreted from activated mast cell granules, it causes the degradation of the extracellular matrix through its proteolytic action. The degradation of the extracellular matrix is an important step in the early phase of angiogenesis [32]. Tryptase, which is mitogenic for endothelial cells [33], also promotes the chemotaxis of inflammatory cells that express cytokines (IL-1, IL-6, IL-8, stem cell factor, TNF-α, and other inflammatory mediators) that stimulate endothelial cells [34]. Tryptase can activate MMP-9, which releases angiogenic factors in the extracellular matrix [35]. It is known that human recombinant tryptase is induced by a well-known angiogenic cytokine, namely VEGF secretion [36]. Few studies with small sample sizes investigate mast cell enzymes in the cholesteatoma matrix. Chymase-positive mast cell accumulation was reported in the cholesteatoma matrix in a study including five cholesteatoma cases diagnosed with chronic otitis media [37]. Similarly, we detected chymase-positive mast cell numbers in 35 cases we included in our study, and chymase-positive mast cell accumulation was higher than the tryptase-positive mast cell number. In addition, numbers of both chymase- and tryptase-positive mast cells were significantly higher than in the control tissue. Unlike previous studies [37], our study’s tryptase-positive mast cell count is not low. The small number of cases in the previous study is likely responsible for the difference. In contrast to solid organ tumours, mast cells with chymase-positive stains are higher than those with tryptase-positive stains in cholesteatoma.

The process of angiogenesis is defined as the development of new vessels during the development of new tissues, and embryogenesis is an essential process in the development of tumour growth, invasion, and metastasis, as well as normal physiological processes such as tissue repair and wound healing [16,38]. Studies examining MVD in cholesteatoma are few, and the number of cases in the studies is still very limited. In a study conducted with 14 cases, while the mean microvessel was 5.44/mm^2^ in the control group, it was found to be 22.79 ± 11.2/mm^2^ in cholesteatoma cases [11]. In our study, like in the study above, MVD was significantly higher in the cholesteatoma group compared to the control group; 10.71 ± 4.15/mm^2^ in the control group and 123.26 ± 50.32/mm^2^ in cholesteatoma groups. Like granulation tissue, cholesteatoma exhibits intense vascularisation. Increasing vascularisation seems to be effective in developing cholesteatoma. Our study found the mean of microvessels in the control group and cholesteatoma cases higher than in the study above. It is possible that this difference can be attributed to a variety of factors, including the demographic differences in the control group, the duration of the lesion in the cholesteatoma group, demographic differences between the patients, and the differences in immune system response.

Even though cholesteatomas are benign, they grow to destroy temporal bone, much like malignant tumours, with a mechanism that is still unclarified [39]. Bone resorption of cholesteatoma is affected by the Ki-67 weighted proliferation index [25], epidermal growth factor [40], matrix metalloproteinase-9 [41], bone morphogenic protein [34], and cytokines such as TNF-alpha [42]. A previous study observed a moderately positive correlation between the measured Ki-67 proliferative index in the cholesteatoma epithelium and bone destruction [26].

The effect and mechanism of angiogenesis on bone destruction in cholesteatoma are unclear [12]. Furthermore, although there is evidence of an increase in mast cells in the cholesteatoma matrix [31], no link has been proven between this increase and bone destruction. The subepithelial connective tissue of cholesteatomas [perimatrix] exhibits angiogenesis. The process of angiogenesis facilitates and sustains the migration of keratinocytes into the middle ear cavity, their increased proliferation, and the expansion of cholesteatomas. As a result, angiogenesis is considered one of this disease’s most destructive aspects [25]. In their investigation, Hamed et al. found that fibroblast growth factors positively stained not only the epithelium but also the mast cell membrane, which means that its expression in the mast cell membrane supports its role in bone resorption activity [4]. The results of our study indicated that the Ki-67 proliferative index of the cholesteatoma epithelium, the number of CD117-positive mast cells, the number of chymase-positive mast cells, the number of tryptase-positive mast cells, and the number of microvessels were not statistically significant in comparing the groups with mild bone erosion and advanced bone erosion. Regardless, we found that the mean of all parameters compared in the group with advanced bone erosion was higher than in the group with mild bone erosion. According to our study, the averages for all the markers examined were high in those with advanced bone erosion, suggesting that all these markers may have some effect on bone erosion. The validity of this claim requires further research with a larger sample size.

Mast cells regulate the tumour microenvironment in solid organ tumours, interact with invasive tumour cells, increase tumour angiogenesis, and contribute to invasion and survival [43]. Many of these settings shown to have potential functions as mast cells are capable of secreting, upon appropriate activation by a variety of immune or non-immune stimuli, a wide variety of cytokines (including many chemokines) and growth factors, which may have autocrine, paracrine, local, and systemic effects [44]. Based on the correlation of immunohistochemical markers in our study, CD117 was positively correlated with chymase, tryptase, and MVD. Tryptase was positively correlated with MVD and chymase. A positive correlation was found between chymase levels and MVD. As a whole, except for the Ki-67 proliferative index, the positive correlation of other markers with each other and with MVD suggests that they promote angiogenesis in the cholesteatoma matrix, as well as in solid organ tumours and wound healing. Whether the mechanism for the upregulation of these enzymes is potentiation of each other or whether the specific one initiates the cascade, angiogenesis, and epithelial proliferation is not yet apparent. Our team will continue to work on this issue. It will be possible to determine the treatment goal and histological follow-up of therapy if we understand the mechanisms underlying cholesteatoma development and destruction.

### The Strength of the Study

To the best of our knowledge, this is the first study to examine the effects of Ki67, CD117, tryptase, chymase, and CD34 on cholesteatoma progression and cholesteatoma-related bone loss and damage. In addition, we presented a relatively wide case population in our study.

## 5. Conclusions

CD117-positive mast cells, particularly chymase-positive mast cells, are found in the cholesteatoma matrix and can increase the proliferative capacity of the cholesteatoma epithelium and MVD (angiogenesis) in the cholesteatoma matrix. Thanks to these effects, they may contribute to the formation of cholesteatoma.

Interestingly, the proportion of chymase-positive mast cells in the cholesteatoma matrix is higher than that of tryptase-positive mast cells. In view of the fact that cholesteatoma is a benign condition, we may have detected it.

As a result of the increased proliferation of cholesteatoma epithelium and increased MVD via chymase-positive mast cells and tryptase-positive mast cells in the matrix, bone erosion was exacerbated. Bone erosion was associated with higher levels of Ki-67, CD117, tryptase, chymase, and CD34, suggesting that all these markers contribute to bone erosion.

Although cholesteatoma is a benign condition, its complications can have serious consequences. In treating cholesteatoma, mast cells can be selected as a target in addition to treatments that reduce the proliferative capacity of the epithelium, and treatments that will reduce MVD prevent bone erosion. This study is the first to examine the relationship between all these immunohistochemical markers and bone erosion.

There is, however, a need for larger studies to elucidate the mediators that contribute to the proliferation of epithelium and the enlargement of microvessels by mast cells.

## Figures and Tables

**Table 1:** The antibodies and the features used in the immunohistochemical study.

Antibodies	Clone	Dilution	Incubation Time	Antigen Retrieval	Company
Kİ67	SP6	1:100	15 Min	ER1/10 Min	Thermo Scientific
CD117	Poliklonal	1:50	15 Min	ER2/10 Min	Thermo Scientific
Tryptase	CC1	1:100	15 Min	ER2/30 Min	Thermo Scientific
Chymase	AA1	1:50	15 Min	ER2/20 Min	Thermo Scientific
CD34	QBend/10	1:50	15 Min	ER2/20 Min	Thermo Scientific

CD117: cluster of differentiation 117; ER1: citrate buffer; ER2: EDTA buffer.

**Table 2:** Comparison of Ki67, CD117, tryptase, chymase, and CD34 immunohistochemical evaluations of the patient and control groups.

Antibodies	Cholesteatoma		Control		*p*
	Min–Max	AVERAGE ± SD	Min–Max	AVERAGE ± SD	
Kİ-67	19.00–150.00	41.23 ± 23.19	12.00–18.00	14.43 ± 2.37	<0.001 *
CD117 (/mm^2^)	55.00–442.00	194.80 ± 92.54	50.00–110.00	71.43 ± 19.52	<0.002 *
Tryptase (/mm^2^)	28.00–204.00	91.89 ± 41.68	24.00–46.00	35.57 ± 6.68	<0.003 *
Chymase (/mm^2^)	36.00–236.00	133.14 ± 59.62	35.00–70.00	48.57 ± 12.25	<0.004 *
MVD-CD34 (/mm^2^)	60.00–276.00	123.26 ± 50.32	6.00–17.00	10.71 ± 4.15	<0.005 *

* *p*_adjusted_ < 0.01, Mann—Whitney U Test. min: minimum; max: maximum; SD: standard deviation; CD117: cluster of differentiation 117.

**Table 3:** The levels of Ki67, CD117, tryptase, chymase, and MVD (CD34) according to bone erosion grading.

Antibodies	Score 1 and below		Score 2 and above		*p*
	Min–Max	M ± SD	Min–Max	M ± SD	
Ki-67	19.00–52.00	35.13 ± 10.43	20.00–150.00	43.92 ± 25.76	0.382
CD117 (/mm^2^)	140.00–236.00	179.50 ± 37.30	55.00–442.00	203.54 ± 103.27	0.626
Tryptase (/mm^2^)	65.00–120.00	89.75 ± 23.98	28.00–204.00	94.73 ± 45.41	0.951
Chymase (/mm^2^)	80.00–160.00	108.50 ± 27.87	36.00–236.00	143.23 ± 64.40	0.133
MVD (CD34) (/mm^2^)	60.00–160.00	100.00 ± 33.49	60.00–276.00	132.85 ± 51.98	0.095

*p*_adjusted_ > 0.01Mann—Whitney U Test. min: minimum; max: maximum; SD: standard deviation; CD117: cluster of differentiation 117; MVD: microvessel density.

**Table 4:** Correlation of immunohistochemical markers’ expressions.

Antibodies		Ki-67	CD117	Tryptase	Chymase	CD34
Ki-67	r	1.000	0.149	0.206	0.096	0.159
CD117 (/mm^2^)	r		1.000	0.871*	0.933 *	0.775 *
Tryptase (/mm^2^)	r			1.000	0.837 *	0.681 *
Chymase (/mm^2^)	r				1.000	0.838 *
MVD (CD34) (/mm^2^)	r					1.000

* *p* < 0.01 Spearman Rank Differences Correlation; CD117: cluster of differentiation 117.

## Data Availability

The data presented in this study are available on request from the corresponding author.
